# CircPSD3 aggravates tumor progression by maintaining TCA cycle and mitochondrial function via regulating SUCLG2 in thyroid carcinoma

**DOI:** 10.1038/s41419-025-07856-x

**Published:** 2025-09-09

**Authors:** Yijia Sun, Beinan Han, Jiawei Ge, Zijun Huo, Jin Li, Bo Lin, Xin Du, Yimin Zhang, Haiyan Weng, Shuang Yu, Yanbing Li, Haipeng Xiao, Xiaorong Lin, Shubin Hong

**Affiliations:** 1 Department of Endocrinology, The First Affiliated Hospital of Sun Yat-sen University, Guangzhou, Guangdong 510080 China; 2 Department of Oncology, Sun Yat-sen Memorial Hospital, Sun Yat-sen University, Guangzhou, 510120 China; 3 Department of Gerontology, The First Affiliated Hospital of Sun Yat-sen University, Guangzhou, Guangdong 510080 China; 4 Department of Thyroid Surgery, The First Affiliated Hospital of Sun Yat-sen University, Guangzhou, Guangdong 510080 China; 5 Diagnosis and Treatment Center of Breast Diseases, Shantou Central Hospital, Shantou, China

**Keywords:** Thyroid cancer, Metabolomics

## Abstract

In recent years, there has been a rapid increase in the incidence of thyroid carcinoma (TC). Our study focuses on the regulatory effect of circular RNAs on metabolism of TC, aiming to provide new insights into the mechanisms of progression and a potential therapeutic target for TC. In this study, we identified high expression levels of circPSD3 in TC tissues through RNA sequencing. Papillary thyroid cancer tissue cohorts verified the circPSD3 expression level was positively correlated with larger tumor size. circPSD3 promoted the proliferation of TC cells and reduced apoptosis both in vitro and in vivo. Proteomics and metabolomics suggested that circPSD3 might play a crucial role in regulating the tricarboxylic acid (TCA) cycle. Specifically, circPSD3 acted as a miR-338-5p sponge to upregulate SUCLG2, an enzyme of the TCA cycle, which accelerates the conversion of α-ketoglutarate (α-KG) to succinate. Knockdown of circPSD3 disrupts the TCA cycle and impairs mitochondrial function, resulting in decreased membrane potential and aerobic respiration rate. The reduction in mitochondrial function resulted in the inhibition of proliferation and initiation of mitochondria-mediated apoptosis.

## Introduction

Thyroid carcinoma (TC) is the most prevalent endocrine malignancy [1]. Most thyroid carcinomas originate from follicular cells, with papillary thyroid carcinoma (PTC) being the most common subtype, constituting 85% of TC cases. Despite the generally favorable prognosis of PTC, clinicopathological features such as lymph node metastasis (37%), extrathyroidal extension (24.8%), and distant metastasis (4.1%) significantly affect TC outcomes [2]. Notably, anaplastic thyroid carcinoma (ATC) is recognized as the most aggressive subtype, with a median survival time of six months [3]. Therefore, a deeper understanding of TC progression mechanisms is essential for developing novel treatments and improving patient prognosis.

Metabolic reprogramming is widely recognized as a hallmark of malignancy. A major metabolic characteristic of tumor cells is their heightened glycolytic activity, even under aerobic conditions, known as the “Warburg effect” [4]. However, the tricarboxylic acid (TCA) cycle, serving as a central pathway for metabolism and oxidative phosphorylation, also plays a pivotal role in cancer development and progression. For instance, elevated carbon flux through the TCA cycle has been observed in non-small cell lung cancer tissues compared to benign lung tissue in vivo using intraoperative ^13^C-glucose infusions [5]. Additionally, lung metastatic tumors of triple-negative breast cancer exhibited higher TCA flux compared to both primary tumors and healthy tissues [6]. These findings suggest that the TCA cycle contributes to cancer development and progression. Although cancer cells typically convert glycolytic pyruvate into lactate, they often activate alternative pathways—such as glutamine and fatty acid metabolism—to fuel the TCA cycle. This overactivation leads to distinct metabolic and epigenetic alterations associated with tumor progression [7]. In glioma, the proto-oncogene MYC, a critical regulator of glutaminolysis, upregulates both glutamine transporters and glutaminase to activate glutamine metabolism and sustain the TCA cycle [8]. Collectively, these findings highlight the active and crucial role of the TCA cycle in tumor progression across several cancer types, although its role in TC remains to be determined.

Over the past decade, circular RNAs (circRNAs) have emerged as a large class of primarily non-coding RNA molecules, characterized by covalently closed loops produced by back-splicing of pre-mRNA [9]. CircRNAs may function through diverse mechanisms, such as microRNA sponging, protein interactions, and protein translation [10]. Previous studies have shown that circNDUFB2 functions as a protein scaffold to activate antitumor immunity [11], while circZNF609 enhances cell proliferation, metastasis, and stemness by sponging miR-15a-5p/15b-5p [12]. Additionally, circRNAs have been shown to regulate the cell cycle, induce apoptosis and autophagy, promote angiogenesis, and reprogram metabolism [13, 14]. In hepatocellular carcinoma (HCC), circMAT2B activated the circMAT2B/miR-338-3p/PKM2 axis to promote cell progression by enhancing glycolysis under hypoxia [15]. CircMBOAT2 promoted cholangiocarcinoma progression by stabilizing PTBP1 to facilitate FASN mRNA cytoplasmic export, thereby altering the lipid metabolic profile [16]. Thus far, the function and mechanism of circRNAs in regulating TC metabolism require further investigation.

Our study demonstrates the upregulation of circPSD3 in TC and its promotion of cell proliferation both in vitro and in vivo. We elucidate that circPSD3 can reprogram the TCA cycle in TC by sponging miR-338-5p to upregulate SUCLG2. Our findings suggest that circPSD3 may serve as a potential therapeutic target aimed at modulating the TCA cycle in TC.

## Materials and methods

### Clinical samples

All PTC and adjacent normal tissues used in this study were obtained from the First Affiliated Hospital of Sun Yat-sen University between 2018 and 2020. The diagnosis of PTC was confirmed by postoperative histopathological examination. The biospecimen collection procedure and research protocol were approved by the Ethics Committee of the First Affiliated Hospital of Sun Yat-sen University.

### Cell lines and cell culture

The cell lines utilized in this study included normal human thyroid epithelial cells (Nthy-ori 3-1), papillary thyroid carcinoma (PTC) cell lines (BCPAP, TPC-1), anaplastic thyroid carcinoma (ATC) cell line (8305 C), and a utility cell line (HEK293T). Nthy-ori 3-1 and BCPAP were generously provided by Professor Haixia Guan from the Department of Endocrinology, Guangdong Provincial People’s Hospital. TPC-1, 8305 C, and HEK293T were obtained from the American Type Culture Collection (ATCC). These cells were authenticated and tested for mycoplasma contamination. All the cells were cultured with specific media according to ATCC instructions.

### Antibodies and sequences

The primary antibodies used in western blotting and RNA immunoprecipitation are listed in Table 1. shRNAs were synthesized by IGE Biotechnology Co., Ltd. (Guangzhou, China). siRNA and miR Inhibitor used in intratumoral injection and probes targeting the junction area of circPSD3 were synthesized by RiboBio Technology Co., Ltd. (Guangzhou, China). Sequences of shRNAs and siRNAs are listed in Table 2. Sequences of miR inhibitors and probes are listed in Table 3.

**Table 1:** Antibodies used in the study.

Antibody	Source	Identifier	Dilution
SUCLG2	Proteintech	14240-1-AP	1:200
OGDH	Proteintech	15212-1-AP	1:300
LaminB1	Abcam	ab16048	1:1000
IgG	Abcam	ab6715	1:100
HA	Abcam	ab9110	1:100

**Table 2:** Sequences of siRNAs and shRNAs.

Name	Sequences
shRNA-circPSD3-1	CTACATGGCCACCAATGCTCTCGAGAGCATTGGTGGCCATGTAG
shRNA-circPSD3-2	TCTACATGGCCACCAATGCCTCGAGGCATTGGTGGCCATGTAGA
siRNA-circPSD3-1	CTACATGGCCACCAATGCT
siRNA-circPSD3-2	TCTACATGGCCACCAATGC

**Table 3:** Sequences of probes and miR inhibitor.

Name	Sequences
circPSD3 probe	GCATTGGTGGCCATGTAGAT
miR-338-5p probe	AACAAUAUCCUGGUGCUGAGUG
miR-338-5p Inhibitor	CACUCAGCACCAGGAUAUUGUU

### Construction of stable cell strains

We constructed two plasmids containing a tetracycline-inducible promoter and two different specific short hairpin RNAs (shRNAs) targeting the junction area of circPSD3. To harvest the lentivirus solution, HEK-293T cells were transfected with the psPAX2 packaging plasmid, PMD2G envelope plasmid, and Tet-sh-circPSD3 using PEI. After 48 h, the supernatant containing the lentivirus was collected and filtered through a 0.4 μm pore size filter. TPC-1 and 8305 C cells were infected with the lentivirus solution containing polybrene (8 μg/mL). After 72 h of incubation, the solution was replaced with complete medium containing puromycin (2–4 μg/mL) for selection. The shRNA targeted the junction area of circPSD3 48 h after doxycycline treatment.

### CircRNA sequencing

The circRNA sequencing for PTC and adjacent tissues was conducted by Liebing Biomedical Technology Co., Ltd. (Shanghai, China). Differential expression analysis of circular RNAs was performed using the edgeR package.

### RNase R treatment

Total RNA was extracted and mixed with ribonuclease R (RNase R) at 37 °C for 20–30 min to digest linear RNA. The RNase R digestion system consisted of 2 μg RNA, 2 U RNase R, 2 μL 10× buffer, and DEPC water adjusted to a final volume of 20 μL. The control group did not include RNase R, while all other components remained the same. After digestion, samples were subjected to 85 °C for 10 min to inactivate the enzyme. RT-qPCR was conducted to assess the levels of linear and circular RNA, respectively.

### Nuclear and cytoplasmic RNA extraction

The nuclear/cytoplasmic RNA fraction was isolated using the PARISTM Kit (Thermo Fisher, AM1921) following the manufacturer’s instructions. Briefly, 1 × 10^7^ adherent cells were collected and washed once with ice-cold PBS. The cells were then incubated with 300 μL separation buffer for 10 min. After centrifugation, the supernatant was collected as the cytoplasmic fraction, while the pellet was retained as the nuclear fraction for subsequent RNA extraction. An equal volume of lysis buffer and 100% ethanol was sequentially added to each fraction, followed by filtration through the provided column to capture RNA on the filter core. After washing the column with wash buffer three times, RNA was eluted by adding elution buffer to the center of the filter core. The obtained nuclear/cytoplasmic RNA was directly used for subsequent RT-qPCR analysis. GAPDH and U3 were employed as internal controls for cytoplasmic and nuclear fractions, respectively, serving as standards for assessing the degree of nuclear-cytoplasmic separation. Primer sequences are listed in Table 4.

**Table 4:** Primers used in qRT-PCR.

DNA/RNA	Forward primer	Reverse primer
circ0004458(circPSD3)	CCATTGCTTCACAAGATGGA	TTTCTGGGGTCTCCTTTTCC
circ0042823	TCTCCTCTAATGACAGGCGATCT	TACTTTCCAGTCTTACAGCCAGC
circ0052001	AGCATGCTGCAGGACGTA	TGTAGATGGGAAGCTGCTTGTA
circ0000842	GCCCTTGCAGAGAAAGAGCATAA	GCCTCCACTATAACTGGTGCAAA
circ0035818	AAGTTACAACACAGATGCCACAG	TCCTCTAGCTCCTCATAGGTTGG
PSD3	GAGTCCATTGCCTTACCTGTG	TCAACACCCTCATTTACCCCTT
GAPDH	AGGTCGGAGTCAACGGATTT	TGACGGTGCCATGGAATTTG
U3	TTCTCTGAGCGTGTAGAGCACCGA	GATCATCAATGGCTGACGGCAGTT
β-actin	GGGAAATCGTGCGTGACATTAAG	TGTGTTGGCGTACAGGTCTTTG
miR-188-3p	GTGGAGCTCCCACATGCAG	GAAAGAAGGCGAGGAGCAGATC
miR-188-5p	GGAGTGGCATCCCTTGCATG	GAAAGAAGGCGAGGAGCAGATC
miR-338-5p	GGGGGATGGTAACAATATCCTGGT	GAAAGAAGGCGAGGAGCAGATC
miR-513a-3p	GGGTGGAGGGTAAATTTCACCTTTC	GAAAGAAGGCGAGGAGCAGATC
miR-548c-3p	GGGAGGGTGGCAAAAATCTCAATT	GAAAGAAGGCGAGGAGCAGATC
miR-885-5p	GTGGAGGGTCCATTACACTACC	GAAAGAAGGCGAGGAGCAGATC

### RNA fluorescence in situ hybridization (FISH)

Cells were seeded onto dishes for confocal microscopy in advance. After washing with PBS, cells were fixed with 4% paraformaldehyde for 10 min, followed by permeabilization for 5 min. Pre-hybridization buffer was applied for 30 min at 37 °C for blocking, and hybridization solution was then prepared by diluting probes to 0.5 μM and incubating overnight at 37 °C. On the following day, the hybridization solution was discarded, and sequential washes with hybridization wash buffer were performed to remove unhybridized probes. DAPI staining solution was used to stain the cell nuclei after washing. Samples were stored at 4 °C with an anti-fading agent for further observation.

### Cell proliferation assays (EdU Assay & CCK-8 Assay)

Cell proliferation rate and cell viability were detected using the Cell-Light EdU Kit (RiboBio, C10310-1) and the Cell Counting Kit-8 (Ape×Bio, K1018). For the EdU assay, treated cells were seeded into a 96-well plate overnight. On the next day, cells were incubated in complete medium containing EdU reagent (1:500) for 2 h, then subjected to Apollo staining and Hoechst 33342 staining according to the manufacturer’s instructions. Fluorescence images were taken under a 5x objective using a fluorescence microscope. EdU positive cells (%) were calculated as (EdU-positive cells / DAPI-positive cells) × 100%. For the CCK-8 assay, cells were treated and seeded into four 96-well plates. After cells were incubated in complete medium containing CCK-8 reagent (10%) for 2 h, the absorbance at 450 nm was measured to represent cell viability at 0, 24, 48, and 72 h. The procedure was carried out for four days at 24-h intervals.

### Cell apoptosis assays

The apoptosis rate was measured using the Annexin V-FITC/PI Apoptosis Kit (Elabscience, E-CK-A211). Cells were seeded in a 6-well plate and treated for 72 h before harvesting. Both cells and cell debris in the supernatant were collected in the same tube, washed once, and then resuspended in 200 μL binding buffer. Cells were stained with Annexin V/PI following the manufacturer’s instructions and then analyzed using fluorescence-activated cell sorting (FACS). FACS was performed using a NovoCyte Flow Cytometer (Agilent Technologies, USA) and analyzed with NovoExpress software. The apoptosis rate was calculated as the proportion of Annexin V + /PI+ and Annexin V + /PI- cells, representing the sum of cells in early and late apoptosis.

### Animal experiments

The study was approved by the Institutional Animal Care and Use Committee (IACUC), Sun Yat-Sen University (SYSU-IACUC-2023-000896). Cells were harvested, washed, resuspended in PBS, and then mixed with Matrigel (Corning, 356234) at a ratio of 1:1. Female BALB/c nude mice at four weeks of age were randomly divided into three or four groups, each group with 6 mice. Each mouse was injected subcutaneously in the back with 10^7^ cells in 100 μL cell suspension. After the volume reached 100 mm^3^, mice were randomly assigned to different groups. The siRNA and miRNA inhibitors were administered into established tumors by multi-point injection every three days at a dosage of 2 nmol per mouse. Tumor volume was measured before each administration using callipers, and the volume was estimated according to the formula: 0.5 × length × width^2^. According to the animal use protocol approved by IACUC, mice were euthanized by cervical dislocation before the maximal tumor size reached 2000 mm^3^ or maximum tumor diameter reached 2 cm. Tumors were dissected, photographed, and weighed. Tumors were fixed in 4% paraformaldehyde for subsequent embedding in paraffin and sectioning.

### Proteomics

Proteomic sequencing was carried out after knocking down circPSD3 in TPC-1 and 8305 C cell lines, with three replicates in each group. Twelve protein samples were subjected to protein mass spectrometry analysis by Novogene Co., Ltd. (Beijing, China). Differential expression analysis of proteins was conducted using the limma package, followed by KEGG pathway enrichment analysis using the clusterProfiler package.

### Metabolomics

Cells were seeded onto 6 cm dishes in advance. Metabolomic sequencing was carried out after knocking down circPSD3 in TPC-1 and 8305 C cell lines, with four replicates in each group. After washing twice with cold PBS, cells were rapidly frozen in liquid nitrogen. Subsequently, samples were fixed in 800 μL methanol at –80 °C for 30 min. Next, the mixture was scraped, transferred to EP tubes, and mixed with an equal volume of acetonitrile. After centrifugation at 4 °C, the supernatant was collected and immediately subjected to LC-MS analysis using the 1290 Infinity II liquid chromatography-mass spectrometry system (Agilent Technologies, USA). Metabolites were standardized based on internal standards, and integrated analysis was performed using MassHunter quantitative software.

### Oxygen consumption rate measurement

The oxygen consumption rate (OCR) was measured using the Seahorse XF96 Analyzer (Agilent Technologies, USA). Cells were seeded into Seahorse XF cell culture microplates at equal quantities (15,000 cells/well for TPC-1 and 20,000 cells/well for 8305 C) one day prior to the experiment to ensure consistent cell density and 90–100% fusion across groups. The complete medium was discarded, and the cells were incubated in 180 μL assay medium for 45 min in a non-CO2, 37 °C incubator. Meanwhile, the reagents were diluted with assay medium, and Oligomycin, FCCP, and Rotenone/Antimycin A were loaded into cartridge ports A, B, and C, respectively. The final concentrations of compounds are Oligomycin (1.5 μM), FCCP (0.5 μM), Rotenone/Antimycin A (0.5 μM/0.5 μM) in every cell. The assay was run according to the template. After the program, we lysed and quantified the protein of remaining cells in the plate. The protein level of every well is used for the following normalization process before analyzing. All reagents and supplies required were purchased from Agilent Technologies, Inc. (USA).

### Mitochondrial membrane potential detection

Mitochondrial membrane potential (MMP) was measured using the MitoProbe™ JC-1 Kit (Thermo Fisher, M34152). JC-1 exhibits potential-dependent accumulation in mitochondria, indicated by a fluorescence emission shift from green ( ~ 529 nm) to red ( ~ 590 nm). Mitochondrial depolarization is indicated by a decrease in the red/green fluorescence intensity ratio. After the cells were digested and gently washed with warm PBS, 10^6^ cells were collected and incubated at 60 °C for 1 min as a positive control group. Cells from the positive control group and other treated groups were incubated with JC-1 solution or blank solution at 37 °C for 30 min. JC-1 was added in 1 mL complete medium at a 1:1000 dilution. After incubation, the cells were washed twice with warm PBS and resuspended in the same solution for FACS. MMP was determined by the polymer/monomer ratio.

### NAD + /NADH measurement

The NAD + /NADH ratio was measured using the NAD + /NADH Ratio Assay Kit (Beyotime, S0175) according to the manufacturer’s manual. Briefly, the intracellular NAD + /NADH fraction was extracted and divided into two groups: half of the sample was used to determine the total concentration of NAD+ and NADH, and the other half (treated at 60 °C for 30 min to resolve the NAD+ fraction) was used to detect the concentration of NADH. The absorbance at 450 nm was measured to calculate the concentration and NAD + /NADH ratio according to the standard curve.

### Metabolite supplementation

Metabolite rescue was conducted by adding α-ketoglutarate (Sigma, K1128) and succinate (Sigma, PHR1418) to complete medium at concentrations of 4 mM and 2 mM, respectively. The conditional medium was adjusted to a neutral pH.

### RNA immunoprecipitation

Cells were transfected with HA-tagged Ago2 plasmid prior to RNA immunoprecipitation. The cells were harvested, centrifuged, and re-suspended in 100 μL IP lysis buffer supplemented with protease and RNase inhibitors for 5 min. Subsequently, the lysate was centrifuged, and the supernatant was transferred to a new EP tube. Ten percent of the supernatant was reserved as an input control and stored at -80 °C. The remaining supernatant was divided into two equal parts: one part was incubated with IgG control antibody, and the other part with HA antibody, both at 4 °C for 2 h. After this incubation, protein A/G agarose beads were added and incubated overnight. The following day, the beads were washed with PBST on a magnetic rack to remove unbound complexes. The immunoprecipitated RNAs were extracted using the TRIzol method for subsequent RT-qPCR analysis.

### RNA pull-down

Cells were transfected with the circPSD3-overexpression plasmid in advance. Forty-eight hours later, the cells were harvested and lysed in IP lysis buffer for 5 min, followed by centrifugation at 4 °C at 13,000 rpm for 10 min. The supernatant was transferred to a new EP tube. Next, NC probe/biotin-labeled circPSD3 probe/biotin-labeled miR-338-5p probes were mixed with the supernatants separately and incubated overnight at 4 °C with rotation. Streptavidin magnetic beads were then added and incubated at room temperature for 2 h with rotation. Afterward, the beads were washed with PBST on a magnetic rack to remove unbound RNA complexes. RNA was extracted using the TRIzol method for subsequent RT-qPCR analysis.

### Dual-luciferase reporter assay

Wild-type and mutant fragments of the SUCLG2 3’UTR were constructed into the pmirGLO vector. The constructed reporter plasmids and miR-338-5p mimics or NC were then co-transfected into 293 T cells. After 48 h of incubation, the luciferase activity was quantified using the Dual-Luciferase® Reporter Assay System (Promega, E1910). The firefly to Renilla luciferase ratios were calculated. MiR-338-5p mimics and miR-NC were synthesized by RiboBio Technology Co., Ltd. (Guangzhou, China).

### HE, IHC and RNA in situ hybridization

PTC tissues and mouse xenograft tumors were fixed with 4% paraformaldehyde, embedded in paraffin, and sectioned. All paraffin sections were deparaffinized and hydrated using xylene and ethanol. For IHC staining, after immersion in boiling EDTA unmasking solution (Solarbio, C1034) for antigen retrieval and subsequent blocking in 20% goat serum, the slides were incubated at 4 °C overnight with a primary antibody against Ki67. The following day, the slices were incubated with a secondary antibody, and the result was detected using a 3,3′-diaminobenzidine (DAB) peroxidase substrate kit (Dako, K5007). For HE staining, sections were stained with eosin and hematoxylin solution only. After dehydration and clearing, the slices were sealed with neutral resin and prepared for subsequent observation under a microscope. RNA in situ hybridization was conducted using an enhanced sensitive in situ hybridization detection kit (BOSTER, MK1030), following the manufacturer’s instructions. CircPSD3 junction site-targeting/non-targeting labeled probes conjugated to digoxin were synthesized by Geneseed Biotech, Co., Ltd. (Guangzhou, China). The slides were hybridized with probes (0.5 μg/mL) at 40 °C overnight. To estimate the level of circPSD3, three areas from each slide were randomly selected, and the positive-staining area [1–4] and positive-staining intensity (0-3) were scored. The total staining score was calculated by multiplying the staining area by the staining intensity. The scoring process was performed independently by two researchers.

### Caspase-3 activity assay

Caspase-3 activity was measured using the Caspase-3 Activity Assay Kit (Beyotime, C1116) following the manufacturer’s instructions. Cells were lysed in 100 μL lysis buffer for 15 min, and the supernatant was incubated with 10 μL Ac-DEVD-pNA (2 mM) at 37 °C for 2 h. During this period, caspase3 will convert Ac-DEVD-pNA into a yellow formazan product of p-nitroaniline (pNA). The resulting p-nitroaniline (pNA) was quantified by absorbance at 405 nm. Caspase-3 activity was calculated based on a standard curve and normalized to total protein concentration determined by the Bradford assay.

### Statistical analysis

All experimental data were obtained from at least three independent trials. Before analyzing, all the data has been under normal distribution test (Shapiro-Wilk test). For normally distributed data, we chose *t*-tests (two-sided) in GraphPad Prism 8.0 software, and presented the data as mean ± SD. For non-normally distributed data, we chose Wilcoxon signed-rank test, and presented the data as median(95%CI). The correlation was analyzed using the Spearman’s rank correlation test. A *P* value of 0.05 was considered statistically significant.

## Results

### circPSD3 is up-regulated in thyroid carcinoma

**Fig. 1:**
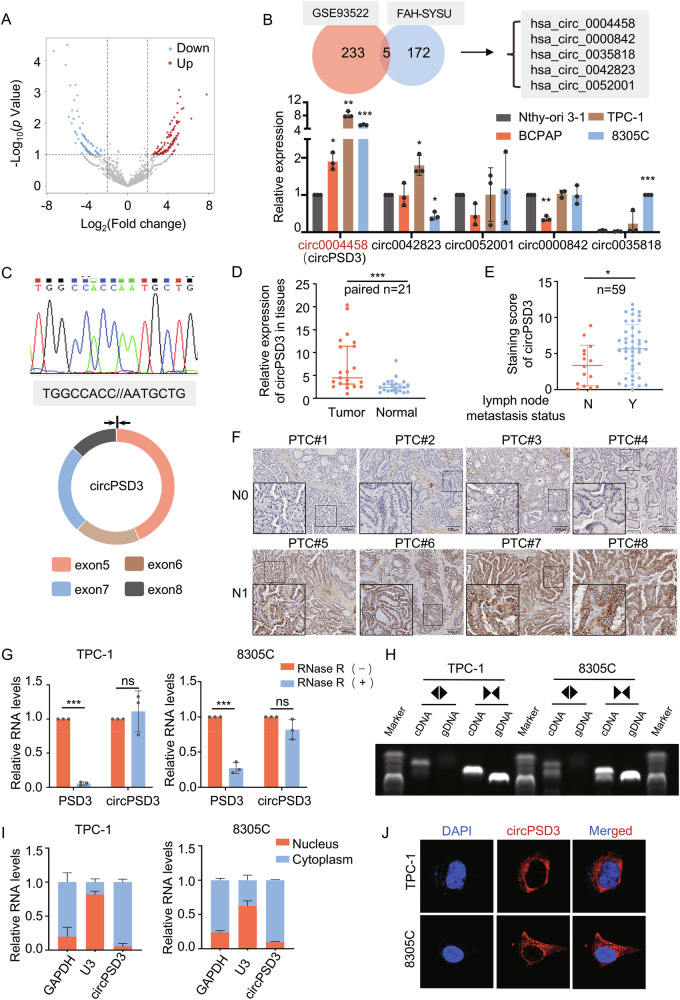
circPSD3 Up-regulation in Thyroid Carcinoma. **A** Volcano plot displaying differentially expressed circular RNAs between 5 PTC tissues and adjacent normal thyroid tissues. **B** (Upper panel) Venn diagram showing a set of 5 upregulated circular RNAs in 2 PTC databases. (Lower panel) Relative expression levels of the 5 circular RNAs in thyroid epithelial cell line (Nthy-ori 3-1) and TC cell lines (TPC-1, BCPAP, 8305 C). **C** (Lower panel) Schematic circular structure of circPSD3 and its junction sequence, verified by PCR and Sanger sequencing (Upper panel). **D** Upregulated levels of circPSD3 in PTC tissues were verified in our validation cohort (*n* = 21). **E**, **F** RNA hybridization in situ illustrating different expression levels of circPSD3 in thyroid carcinoma tissues with or without lymph node metastasis (**F**). Staining score of circPSD3 was calculated, indicating a higher circPSD3 expression level in tissues with lymph node metastasis (**E**). **G** Relative RNA levels of linear PSD3 and circPSD3 after treatment with or without RNaseR. **H** Electropherogram displaying the PCR amplification products by divergent primers or convergent primers. **I** Relative RNA levels of circPSD3 in nuclear and cytoplasmic fractions from TPC-1 and 8305 C. **J** Subcellular localization of circPSD3 in TPC-1 and 8305 C detected by FISH. The experiments were repeated 3 times independently. Normally distributed data are presented as the mean ± S.D, non-normally distributed data are presented as median (95%CI). cDNA complementary DNA, gDNA genomic DNA. *P < 0.05; **P < 0.01; ***P < 0.001; ns no significance.

We performed RNA sequencing on five pairs of PTC and adjacent normal thyroid tissues, identifying 177 upregulated and 149 downregulated circRNAs, as shown in the volcano plot (Fig. 1A). Five upregulated circRNAs from RNA sequencing were identified by re-analyzing the public database of GSE93522 to screen for the most representative circRNAs in thyroid cancer (Fig. 1B, upper). Based on the expression levels of these five circRNAs in the normal thyroid cell line Nthy-ori 3-1 and three TC cell lines, circPSD3 was found to be the most significantly upregulated circRNA, consistent with the sequencing results (Fig. 1B, lower). CircPSD3 is generated by back splicing of exons 5–8 of PSD3 (Fig. 1C) and was upregulated in PTC tissues relative to adjacent normal thyroid tissues in our clinical cohort (Fig. 1D). Subsequently, we investigated the relationship between circPSD3 expression and clinicopathological features in our cohort. The data showed that circPSD3 expression level was positively correlated with tumor size (Supplementary Fig. 1A). Furthermore, there was a probable positive trend between lymph node metastasis status and circPSD3 expression level, while gender, age, multifocality, and extrathyroidal extension appeared to have no correlation with circPSD3 level (Supplementary Fig. 1B, C). To further validate this correlation, we performed RNA in situ hybridization in an independent cohort to assess circPSD3 expression. The results indicated that tumors with lymph node metastasis exhibited a higher expression level of circPSD3 (Fig. 1E, F). In contrast to its linear isoform, circPSD3 exhibited resistance to RNase R digestion in TC cells 8305 C and TPC-1 (Fig. 1G). Sanger sequencing and DNA gel electrophoresis demonstrated that the back-splice junction of circPSD3 was amplified using divergent primers (Fig. 1H). In accordance with these results, the properties of circPSD3 as a circular RNA were validated. Additionally, nucleoplasm separation followed by qPCR and FISH analyses of circPSD3 localization revealed that circPSD3 was predominantly localized in the cytoplasm, with a minor presence in the nucleus (Fig. 1I, J).

### circPSD3 promotes cell proliferation and inhibits cell apoptosis in thyroid carcinoma

**Fig. 2:**
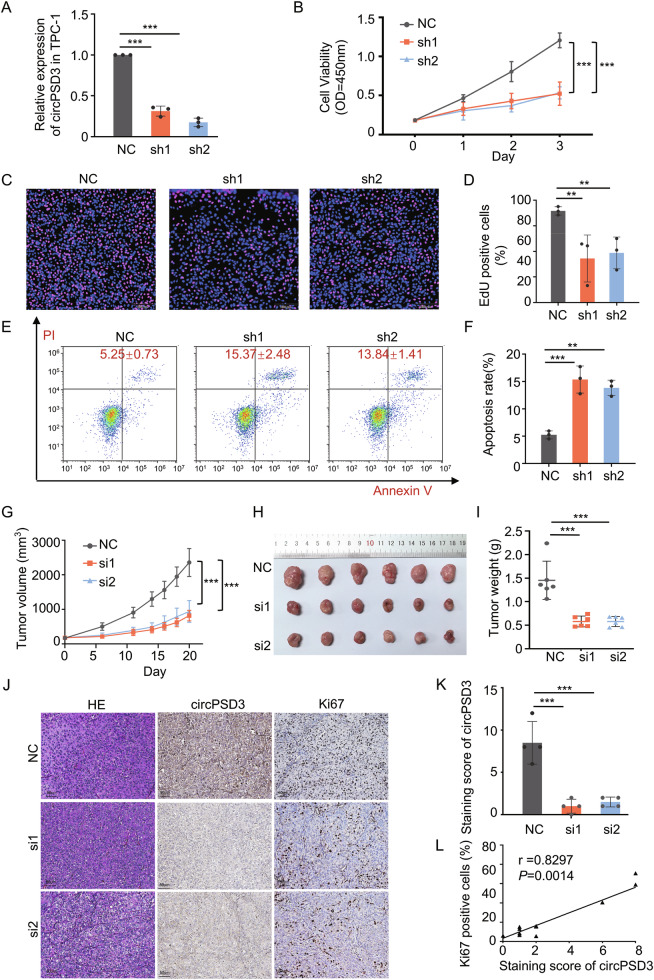
circPSD3 enhances cell proliferation and inhibits apoptosis in thyroid carcinoma. **A** Efficiency of circPSD3 knockdown in TPC-1. **B** Cell viability detected by CCK-8 assay after circPSD3 knockdown in TPC-1. **C** Representative EdU fluorescence images demonstrating the inhibitory effect of circPSD3 on proliferation in TPC-1. **D** Statistical results of EdU proliferation assay in TPC-1. **E** Apoptotic cells analyzed by flow cytometry in TPC-1. **F** Statistical results of apoptotic cell rate detected by flow cytometry in TPC-1. **G**–**I** Subcutaneous xenograft tumors after multiple intratumoral injections with siNC, sicircPSD3-1, and sicircPSD3-2. Tumor growth curves (**G**), gross appearance of tumors taken on the same scale (**H**), and tumor weight analysis (**I**) of TPC-1. **J** Representative images of TPC-1 xenograft tumors by HE, FISH, and Ki67 staining. **K** Efficiency of circPSD3 knockdown in TPC-1 xenograft tumors by circPSD3 staining. **L** Correlation between circPSD3 and Ki67 expression in TPC-1 xenograft tumors. The experiments were repeated 3 times independently. Data are presented as the mean ± S.D. NC negative control group, sh1 sh-circPSD3-1 group, sh2 sh-circPSD3-2 group. *P < 0.05; **P < 0.01; ***P < 0.001.

To investigate the function of circPSD3 in TC, we selected the TPC-1 and 8305 C cell lines, which exhibited the highest expression levels. We employed two short hairpin RNAs to create stable knockdown of circPSD3. The sh-circPSD3 groups demonstrated a notable decrease in circPSD3 expression compared to the NC group (Fig. 2A and Supplementary Fig. 2A). The downregulation of circPSD3 was correlated with reduced cell viability, detected by the CCK8 assay (Fig. 2B and Supplementary Fig. 2B) and the EdU assay (Fig. 2C, D and Supplementary Fig. 2C, D). Furthermore, flow cytometry analysis revealed the rates of cell apoptosis increased following the suppression of circPSD3 (Fig. 2E, F and Supplementary Fig. 2E, F). Moreover, sh-circPSD3 significantly suppressed tumor growth in vivo compared to the NC group. (Fig. 2G–I and Supplementary Fig. 2G-I). In the circPSD3 shRNA group, xenograft tumors exhibited significantly reduced circPSD3 expression, accompanied by a decrease in Ki67 levels. (Fig. 2J, K and Supplementary Fig. 2J-K) Correlation analysis further confirmed a positive association between circPSD3 and Ki67 expression levels. (Fig. 2L and Supplementary Fig. 2L). These results indicated that circPSD3 promoted the proliferation of TC cells and reduced apoptosis both in vitro and in vivo.

### circPSD3 regulates the TCA cycle and mitochondrial function via SUCLG2

**Fig. 3:**
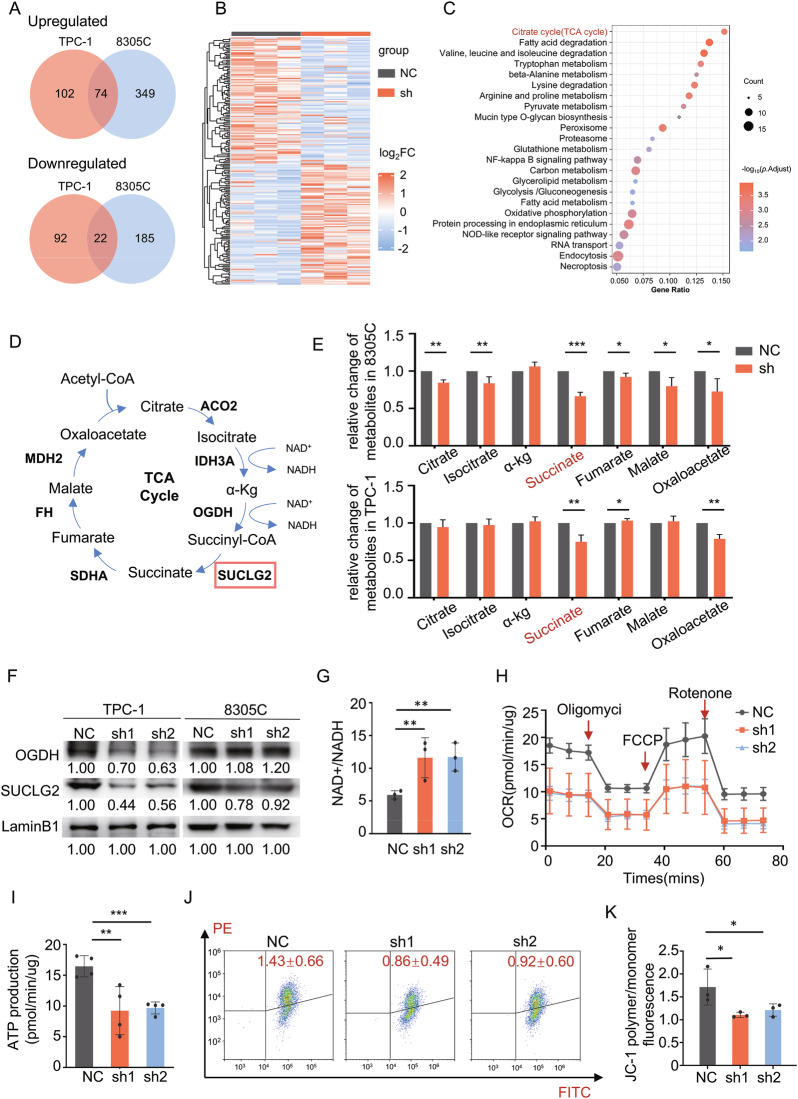
circPSD3 regulation of the TCA cycle and mitochondrial function via SUCLG2. **A** Venn diagram showing the differentially expressed protein profile between the sh-circPSD3 group and NC group detected by mass spectrometry (MS) in TPC-1 and 8305 C. **B** Heatmap of the differentially expressed protein profile between the sh-circPSD3 group and NC group in TPC-1. **C** Kyoto Encyclopedia of Genes and Genomes (KEGG) pathway analysis based on down-regulated expressed proteins in either TPC-1 or 8305 C. **D** Schematic diagram showing the TCA cycle and its key enzymes and intermediate metabolites. Downregulated proteins in both cell lines are highlighted. **E** TCA metabolites were extracted and detected in the sh-circPSD3 group compared with the NC group. **F** Western blotting confirmed the differential expression of key enzymes in proteomics. **G** NAD + /NADH ratio was elevated in the sh-circPSD3 group in TPC-1. **H** OCR was measured by Seahorse XF analyzer in TPC-1, indicating a suppressed maximal respiration and ATP production after circPSD3 knockdown. The data has been normalized on the protein quantification. **I** ATP production from OXPHOS was calculated according to OCR. **J** MMP was measured by flow cytometry using JC-1. Red fluorescence (PE channel) represented polymers and green fluorescence (FITC channel) represented monomers. Knockdown of circPSD3 led to a decrease in the polymer/monomer fluorescence intensity ratio, indicating mitochondrial depolarization. **K** Statistical results of MMP. MMP is calculated as JC-1 polymer/monomer fluorescence ratio. The experiments were repeated 3 times independently. Data are presented as the mean ± S.D. MMP mitochondrial membrane potential, OCR oxygen consumption rate, NC negative control group, sh1 sh-circPSD3-1 group, sh2 sh-circPSD3-2 group. *P < 0.05; **P < 0.01; ***P < 0.001.

Proteomics was employed to evaluate the differentially expressed proteins in TPC-1 and 8305 C cells after circPSD3 knockdown to investigate the potential targets and signaling pathways of circPSD3(Fig. 3A, B and Supplementary Fig. 3A). KEGG analysis indicated that circPSD3 played a pivotal role in multiple metabolic pathways, in which the TCA cycle emerged as the most prominent pathway (Fig. 3C). The differentially expressed protein profile from proteomics revealed a significant downregulation of key enzymes in the tricarboxylic acid (TCA) cycle, including ACO2, OGDH, SUCLG2, and FH, with SUCLG2 being significantly downregulated in both cell lines (Supplementary Fig. 3B).

Metabolomics analysis was also performed to detect changes of intermediate metabolites in both the sh-circPSD3 and NC groups. The results showed a significant decrease in succinate, citrate, and isocitrate levels in the sh-circPSD3 group in 8305 C cells, while succinate and oxaloacetate levels were reduced in the sh-circPSD3 group in TPC-1 cells (Fig. 3D, E). Integrating the findings from proteomics and metabolomics suggested that circPSD3 might play a crucial role in regulating the conversion of α-ketoglutarate (α-KG) to succinate. Consequently, decreased expression of SUCLG2, which facilitates the transformation of α-KG into succinate within the TCA cycle, was observed in the sh-circPSD3 group compared to the NC group in both TPC-1 and 8305 C cells (Fig. 3F and Supplementary Fig. 3C).

An increased NAD + /NADH ratio was another hallmark of the disrupted TCA cycle (Fig. 3G and Supplementary Fig. 3D). Given the significant influence of the TCA cycle on mitochondrial function, our subsequent investigation focused on examining mitochondrial aerobic respiration and mitochondrial membrane potential (MMP). Our study demonstrated that downregulation of circPSD3 impeded mitochondrial aerobic respiration, as measured by the Seahorse assay. (Fig. 3H, I and Supplementary Fig. 3E, F). Additionally, decreased circPSD3 expression resulted in reduced MMP levels, as observed through JC1 staining (Fig. 3J, K and Supplementary Fig. 3G, H).

The reduction in mitochondrial function triggered the activation of caspase-3, resulting in the initiation of mitochondria-mediated apoptosis (Supplementary Fig. 3I). Overall, these findings suggested that inhibiting circPSD3 could reduce the protein expression of SUCLG2, thereby impeding the production of succinate in the tricarboxylic acid (TCA) cycle and suppressing mitochondrial activity, ultimately leading to apoptosis.

### circPSD3 promotes cell proliferation by regulating TCA cycle in thyroid carcinoma

**Fig. 4:**
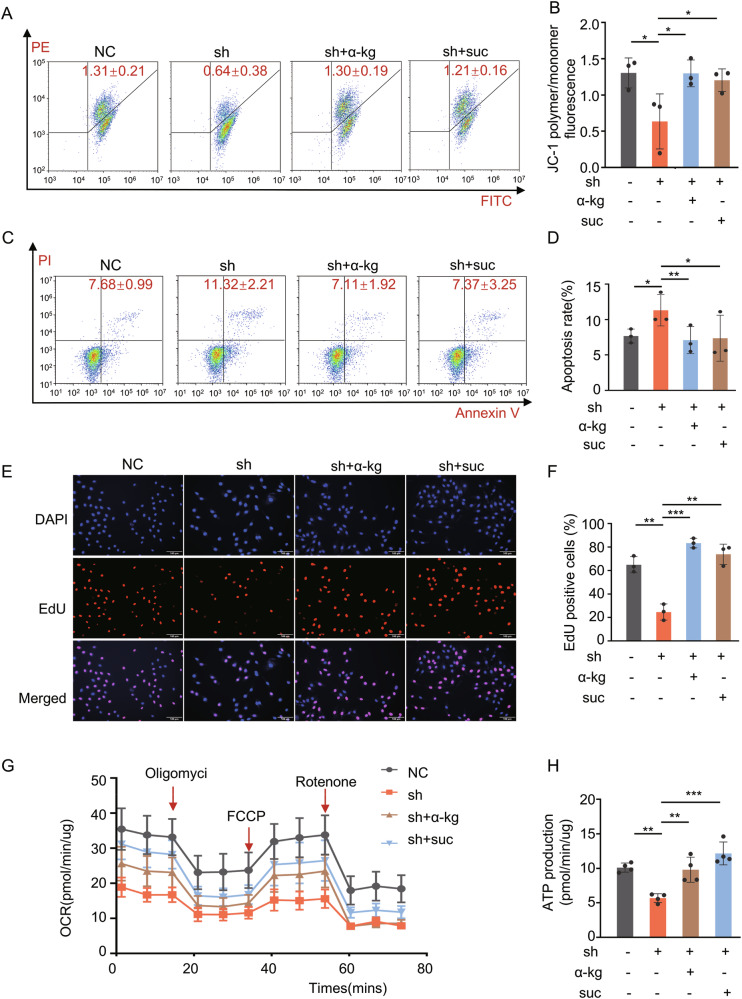
circPSD3 promotes cell proliferation by regulating the TCA cycle in thyroid carcinoma. **A**, **B** Flow cytometry and statistical results demonstrate the decreased MMP in the sh-circPSD3 group, which was rescued by regaining α-ketoglutarate and succinate in TPC-1. **C**, **D** Flow cytometry and statistical results demonstrate the increased apoptotic cell rate in the sh-circPSD3 group, which was rescued by regaining α-ketoglutarate and succinate in TPC-1. **E**, **F** Representative EdU fluorescence images and statistical results demonstrate the inhibitory effect on proliferation of circPSD3-knockdown, which was rescued by regaining α-ketoglutarate and succinate in TPC-1. **G**, **H** The suppressed OCR and decreased ATP production in the sh-circPSD3 group, which was rescued by regaining α-ketoglutarate and succinate in TPC-1. The experiments were repeated 3 times independently. Data are presented as the mean ± S.D. α-kg α-ketoglutarate, suc succinate, NC negative control group, sh sh-circPSD3 group. *P < 0.05; **P < 0.01; ***P < 0.001.

To confirm the role of circPSD3 in cell growth and its impact on the TCA cycle, we supplemented the sh-circPSD3 group with additional α-ketoglutarate and succinate, as these metabolites are up- or down-stream of SUCLG2. Flow cytometry analysis revealed that the decrease in mitochondrial membrane potential (Fig. 4A, B and Supplementary Fig. 4A, B) and the increase in cell apoptosis caused by circPSD3 knockdown were restored with the addition of α-ketoglutarate and succinate (Fig. 4C, D and Supplementary Fig. 4C, D). This rescue effect was consistent with the results of the EdU assay. (Fig. 4E, F and Supplementary Fig. 4E, F). Additionally, the reduced oxygen consumption rate (OCR) and ATP synthesis resulting from circPSD3 knockdown were also rescued after supplementation with α-ketoglutarate and succinate (Fig. 4G, H and Supplementary Fig. 4G, H). These findings suggest that circPSD3 suppression impairs mitochondrial function by disrupting the TCA cycle, thereby inhibiting cell proliferation.

### circPSD3 functions as a sponge of miR-338-5p

**Fig. 5:**
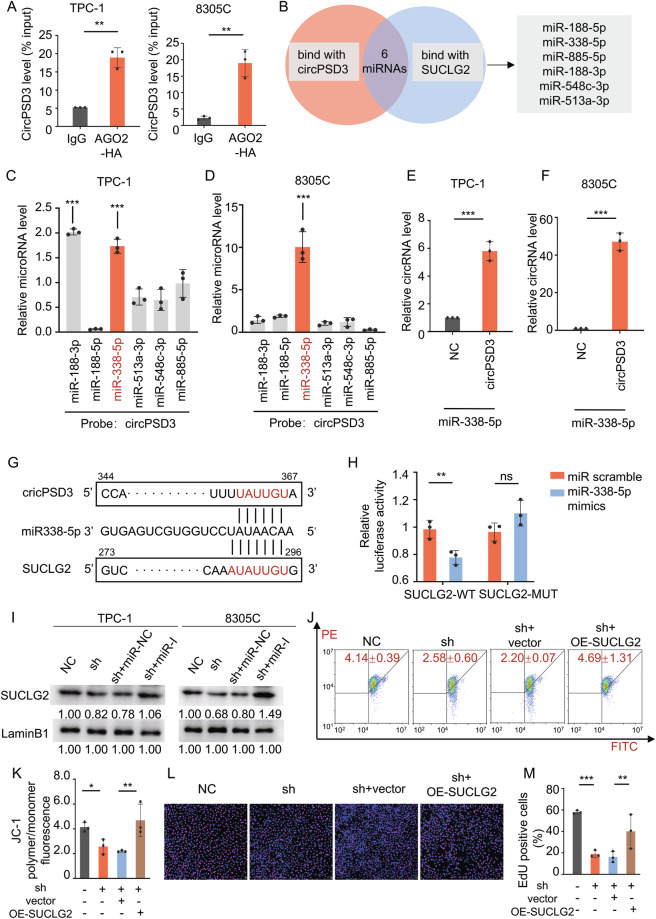
circPSD3 as a Sponge of miR-338-5p. **A** RIP using an anti-IgG antibody or anti-HA antibody followed by RT-qPCR revealed the binding between circPSD3 and AGO2 in TPC-1 and 8305 C. **B** Venn diagram representing the potential binding miRNAs of circPSD3 and SUCLG2 predicted by a bioinformatics website. RNA pull-down assay using a circPSD3 probe followed by RT-qPCR revealed the binding between circPSD3 and miR-338-5p in TPC-1 (**C**) and 8305 C (**D**). RNA pull-down assay using a miR-338-5p probe followed by RT-qPCR confirmed the binding between circPSD3 and miR-338-5p in TPC-1 (**E**) and 8305 C (**F**). **G** Schematic diagram illustrating the motif of miR-338-5p as a link between circPSD3 and SUCLG2. **H** The dual-luciferase assay reports the interaction between miR-338-5p and SUCLG2 mRNA 3ʹUTR motif. **I** Western blotting showed that SUCLG2 downregulation induced by circPSD3 knockdown was reversed by co-treatment with a miR-338-5p inhibitor. **J**, **K** Flow cytometry and statistical results demonstrated that overexpression of SUCLG2 rescued the decreased MMP caused by circPSD3 knockdown in TPC-1. **L**, **M** Representative EdU fluorescence images and statistical results demonstrated the inhibitory effect on proliferation of circPSD3-knockdown was rescued by SUCLG2 overexpression in TPC-1. The experiments were repeated 3 times independently. Data are presented as the mean ± S.D. miR-NC miRNA inhibitor negative control, miR-I hsa-miR-338-5p inhibitor, NC negative control group, sh sh-circPSD3 group. *P < 0.05; **P < 0.01; ***P < 0.001.

As circPSD3 was predominantly located in the cytoplasm, we hypothesized that it functioned as a miRNA sponge to regulate the expression of SUCLG2. Initially, the RNA immunoprecipitation (RIP) assay was conducted to assess its potential role as a miRNA sponge. The anti-HA antibody showed a significant enrichment of circPSD3 compared to the anti-IgG antibody, indicating a binding interaction between AGO2 and circPSD3 (Fig. 5A). Potential target miRNAs of both circPSD3 and SUCLG2 were predicted using the online bioinformatics tool (https://circinteractome.nia.nih.gov/) (Fig. 5B), and six potential miRNAs were identified.

The RNA pull-down assay, using a specific anti-circPSD3 probe, revealed that only miR-338-5p was enriched in both TPC-1 and 8305 C cell lines (Fig. 5C, D). Meanwhile, circPSD3 was significantly enriched using the miR-338-5p probe (Fig. 5E, F). The direct interaction between circPSD3 and miR-338-5p was further confirmed by a dual-luciferase experiment (Fig. 5G, H). Western blot analysis showed that inhibition of miR-338-5p effectively restored the downregulation of SUCLG2 caused by the knocking-down of circPSD3 (Fig. 5I).

To verify SUCLG2 as the downstream target of circPSD3, we performed rescue experiments by overexpressing SUCLG2 after circPSD3 knockdown. The efficiency of SUCLG2 overexpression was confirmed by western blot analysis (Supplementary Fig. 5A). Upregulation of SUCLG2 mitigated the decrease in MMP (Fig. 5J, K and Supplementary Fig. 5B, C) and cell proliferation (Fig. 5L, M and Supplementary Fig. 5D, E) resulting from circPSD3 knockdown. Therefore, it could be inferred that circPSD3 served as a miR-338-5p sponge, influencing the malignant characteristics of thyroid cancer through the modulation of SUCLG2 expression.

### circPSD3 regulates TCA cycle and promotes proliferation by sponging miR-338-5p

**Fig. 6:**
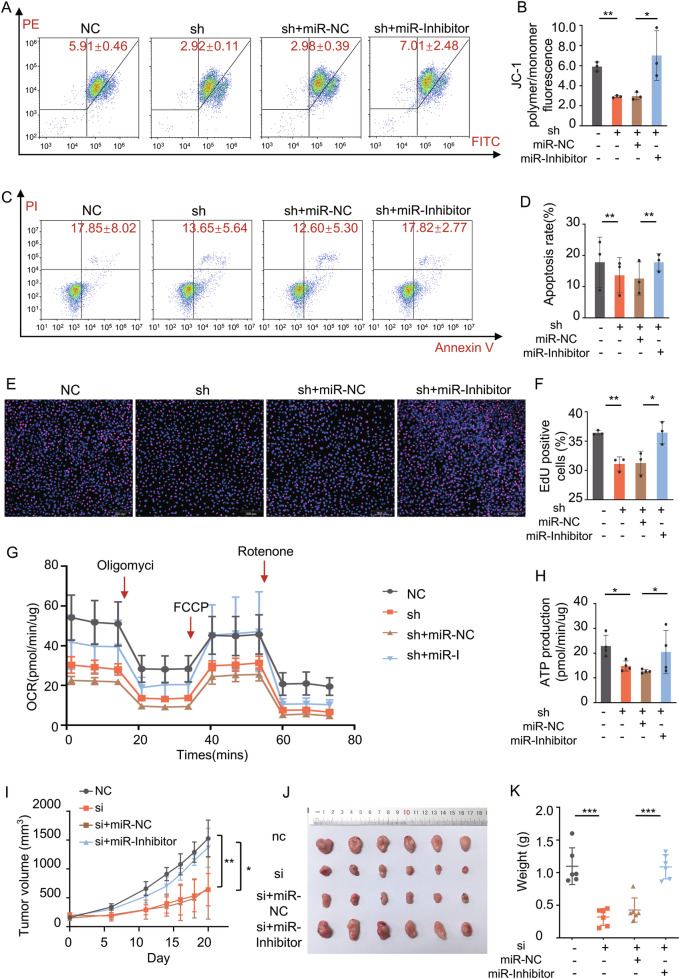
circPSD3 regulation of the TCA cycle and promotion of proliferation by Sponging miR-338-5p. **A**, **B** Flow cytometry and statistical results revealed the miR-338-5p inhibitor reversed the dropping MMP in the sh-circPSD3 group in TPC-1. **C**, **D** Flow cytometry and statistical results revealed the miR-338-5p inhibitor reversed the increased apoptotic cell rate in the sh-circPSD3 group in TPC-1. **E**, **F** Representative EdU fluorescence images and statistical results revealed the miR-338-5p inhibitor reversed the inhibitory effect on proliferation in the sh-circPSD3 group in TPC-1. **G**, **H** The suppressed mitochondrial respiration and ATP production in the sh-circPSD3 group were rescued by the miR-338-5p inhibitor in TPC-1. **I** Subcutaneous xenograft tumors after multiple intratumoral injections with siNC, si-circPSD3, si-circPSD3+NC-inhibitor, si-circPSD3+miR-338-5p inhibitor. Tumor growth curves (**I**), gross appearance of tumors taken on the same scale (**J**), and tumor weight analysis (**K**) of TPC-1. The experiments were repeated 3 times independently. Data are presented as the mean ± S.D. miR-NC miRNA inhibitor negative control, miR-I hsa-miR-338-5p inhibitor, NC negative control group, sh sh-circPSD3 group. *P < 0.05; **P < 0.01; ***P < 0.001.

**Fig. 7:**
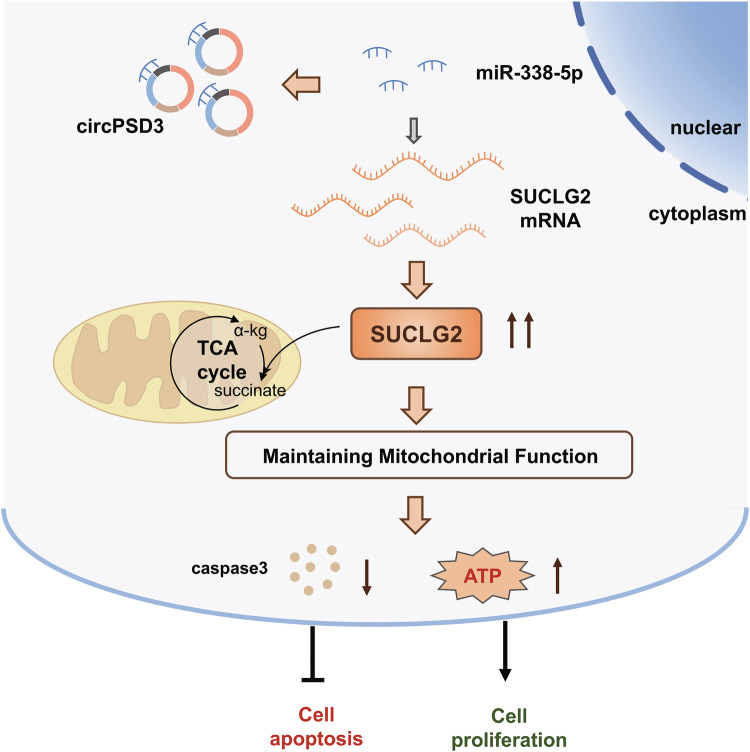
Schematic illustration of the mechanism of circPSD3 in thyroid carcinoma.

To further elucidate circPSD3’s role as a sponge for miR-338-5p in regulating the TCA cycle, we displayed rescue experiments using a miR-338-5p inhibitor. Flow cytometry analysis validated the restored effects of the miR-338-5p inhibitor on mitochondrial damage (Fig. 6A, B and Supplementary Fig. 6A, B) and cell apoptosis (Fig. 6C, D and Supplementary Fig. 6C, D) caused by circPSD3 knockdown. The miR-338-5p inhibitor significantly enhanced mitochondrial function and reversed the increase in apoptosis induced by circPSD3 knockdown. Additionally, the EdU assay results showed that the miR-338-5p inhibitor effectively countered the inhibitory impact of sh-circPSD3 on cell proliferation (Fig. 6E, F and Supplementary Fig. 6E, F). The Seahorse assay yielded similar findings in terms of OCR and ATP generation rate (Fig. 6G, H and Supplementary Fig. 6G, H). Consistent outcomes were observed in nude mice regarding xenograft tumor formation, indicating that the in vivo growth inhibition of subcutaneous tumors was effectively counteracted by the miR-338-5p inhibitor (Fig. 6I–K and Supplementary Fig. 6I-K). Overall, these results suggested that circPSD3 acted as a sponge for miR-338-5p, thereby facilitating the development of malignant characteristics in thyroid cancer (Fig. 7).

## Discussion

Circular RNAs (circRNAs), a class of predominantly non-coding RNA molecules, have been found to exhibit varying expression levels and play significant roles in the development and progression of cancer through various mechanisms [14]. PSD3 (pleckstrin and sec7 domain-containing 3) is an oncogene highly expressed in thyroid cancer (TC), generating multiple circRNAs from distinct exons. Our study focused on circPSD3 derived from exons 5-8 (circBase ID: hsa_circ_0004458) [17]. A previous study reported upregulation of hsa_circ_0004458 in gastric cancer and papillary thyroid carcinoma (PTC), which aligns with our findings showing significant upregulation in TC tissues and cells [18, 19]. Additionally, our study highlighted a positive correlation between its expression level and tumor size as well as lymph node metastatic status, indicating an oncogenic role for circPSD3 in TC by enhancing cancer cell proliferation and suppressing apoptosis.

Proteomics analysis suggests that circPSD3 regulates the tricarboxylic acid (TCA) cycle as one of its downstream pathways. The TCA cycle comprises a series of biochemical reactions in the mitochondrial matrix, providing energy, macromolecules, and redox balance to the cell [7]. Despite the Warburg effect’s assertion that cancer cells primarily generate ATP via glycolysis, evidence supports the notion that some cancer cells utilize oxidative phosphorylation (OXPHOS) as the major means, relying on TCA cycle flux [20, 21]. TC exhibits a very active TCA cycle [22]. Overactivated pyruvate gluconeogenesis and glutamine metabolism replenish intermediate metabolites and fuel the TCA cycle in TC [23, 24]. Besides its catabolic role, the TCA cycle also serves an anabolic function, providing precursors for other biosynthetic processes, including gluconeogenesis, fatty acid, steroids, and amino acids biosynthesis [25]. Our study confirmed that disrupted TCA cycle suppressed NADH and ATP generation via OXPHOS. Furthermore, we observed mitochondrial damage associated with circPSD3 knockdown, characterized by a loss in mitochondrial membrane potential and decreased oxidative respiration rate.

Previous studies have shown that abnormal TCA enzymes in cancer cells lead to impaired mitochondrial function. Mitochondrial membrane potential (MMP) is a critical bioenergetic parameter reflecting mitochondrial condition [26, 27]. Decreased MMP contributes to an early and irreversible step in a cascade of events, including the release of cytochrome c and subsequent activation of caspase-3, ultimately leading to apoptosis via the intrinsic mitochondrial pathway [28, 29]. Our study detected a decrease in MMP and downstream activation of caspase-3, resulting in increased mitochondria-mediated apoptosis associated with circPSD3 knockdown.

Subsequently, we demonstrated that circPSD3 promotes the conversion of α-ketoglutarate (α-KG) to succinate by upregulating SUCLG2. SUCLG2 overexpression and succinate accumulation have been shown to promote tumorigenesis and progression in various malignancies [29]. Additionally, succinate accumulation in tumors stabilizes HIF-1α and activates related oncogenes by inhibiting the activity of prolyl hydroxylases (PHDs) [7]. A recent study indicated that the deletion of SUCLG2 upregulates succinylation levels of mitochondrial proteins and inhibits the function of key metabolic enzymes, impairing mitochondrial function in lung adenocarcinoma cells [30]. Our study observed an overall metabolic disorder in the TCA cycle related to the downregulation of circPSD3 and SUCLG2, characterized by succinate deficiency. The findings from the aforementioned study may provide insights into the overall metabolic disorder observed in our research. These findings suggest that SUCLG2 acts as a potent oncoprotein through several pathways, and targeting the TCA cycle via SUCLG2 may represent a promising therapeutic strategy.

Building on our previous findings that circPSD3 is predominantly located in the cytoplasm and is formed by exons, we have confirmed the circPSD3/miR-338-5p/SUCLG2 regulatory pathway in the progression of TC. Studies have found that circRNAs function by binding to miRNAs in TC [31, 32].

In summary, our study identified that circPSD3 is upregulated and positively correlates with tumor size in TC. CircPSD3 acts as a regulator in the TCA cycle through the circPSD3-miR-338-5p-SUCLG2 axis, maintaining aerobic respiration and stabilizing mitochondrial function, thereby enabling proliferation and preventing mitochondria-mediated apoptosis in TC. Our present study provides insights into the mechanisms of progression and a promising therapeutic target for TC.

## Conclusion

In this study, we identified a high expression level of circPSD3 in TC. Knockdown of circPSD3 disrupted the TCA cycle and caused mitochondrial dysfunction, thereby inhibiting proliferation and inducing apoptosis in TC. Mechanistically, circPSD3 acted as a miR-338-5p sponge to upregulate SUCLG2, an enzyme of the TCA cycle, thereby sustaining the TCA cycle and preserving mitochondrial function.

## Supplementary information

original western blot

supplementary data

## Data Availability

All datasets generated and analyzed during the current study are not publicly available but are available from the corresponding author upon reasonable request.
