# Supplementary material for: CircPSD3 aggravates tumor progression by maintaining TCA cycle and mitochondrial function via regulating SUCLG2 in thyroid carcinoma
Source: Cell Death Dis. 2025 Sep 9;16(1):590. doi: 10.1038/s41419-025-07856-x (PMC12420806; doi:10.1038/s41419-025-07856-x)

Figure 3F (TPC-1)

OGDH

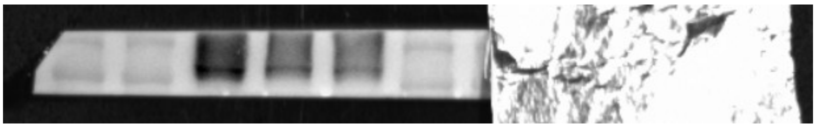

LaminB1

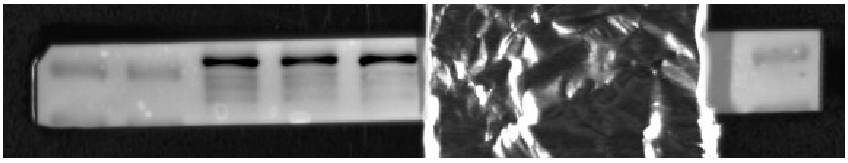

SUCLG2

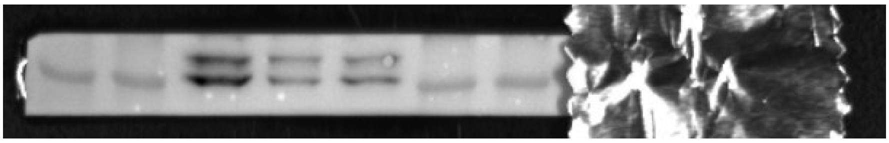

Figure3F (8305C)

OGDH

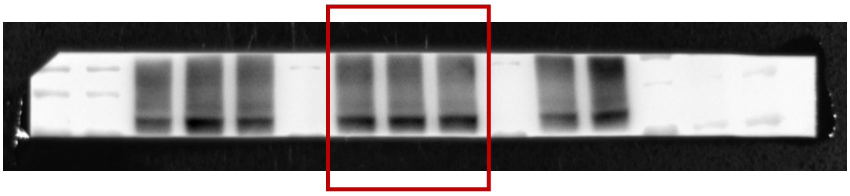

LaminB1

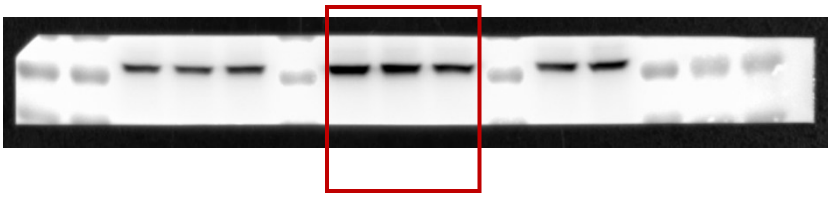

SUCLG2

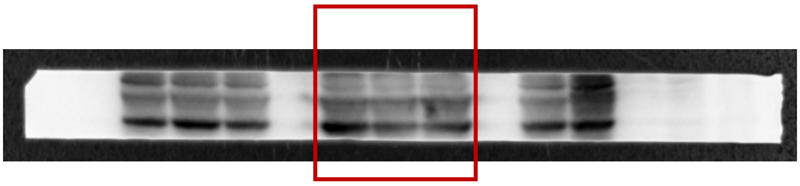

Figure5I

SUCLG2(TPC-1)

SUCLG2(8305c)

LaminB1

(Left: TPC-1)

(Right: 8305C)

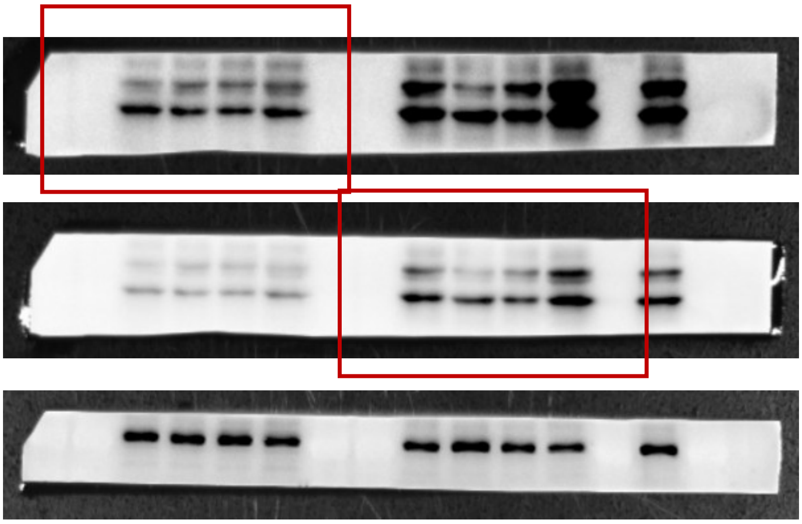

Supplement: Supplementary file 1 — original western blot [file 41419_2025_7856_MOESM1_ESM.pdf]
