# Supplementary material for: CircPSD3 aggravates tumor progression by maintaining TCA cycle and mitochondrial function via regulating SUCLG2 in thyroid carcinoma
Source: Cell Death Dis. 2025 Sep 9;16(1):590. doi: 10.1038/s41419-025-07856-x (PMC12420806; doi:10.1038/s41419-025-07856-x)
Supplement: Supplementary file 2 — supplementary data [file 41419_2025_7856_MOESM2_ESM.pdf]

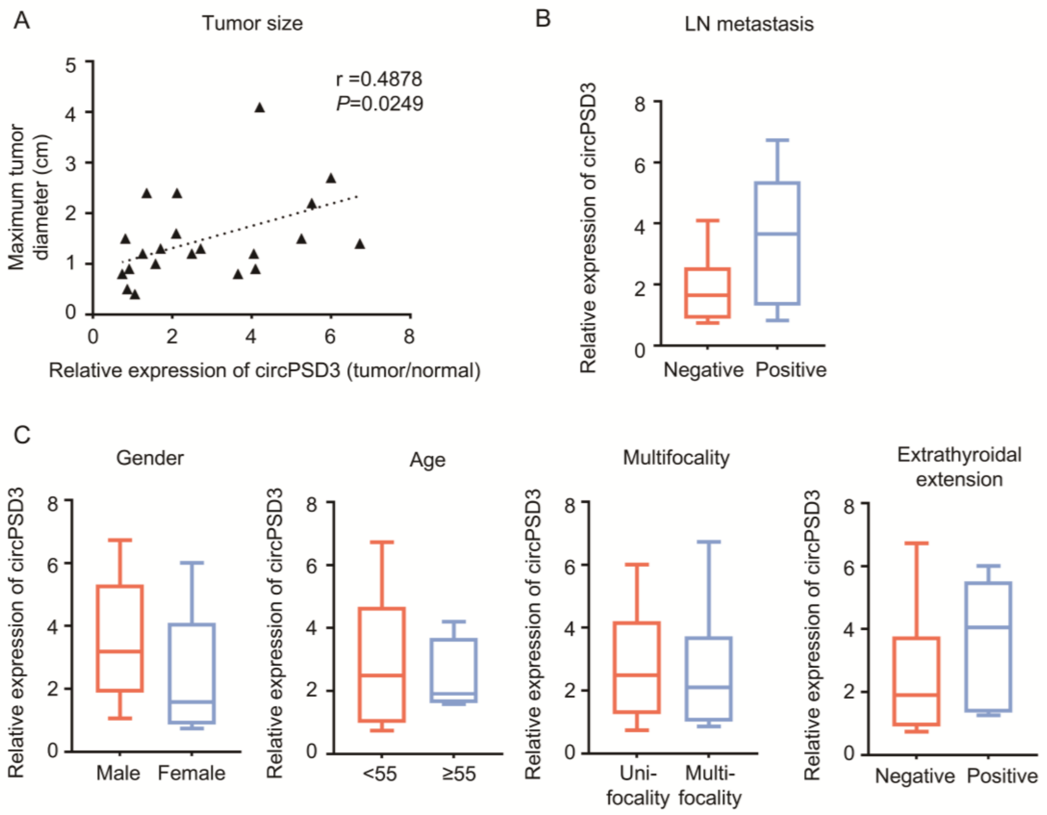

Supplementary Figure 1: Correlation between Relative Expression Level of circPSD3 and Clinicopathological Features.

(A) The correlation between the relative expression level of circPSD3 and the Maximum Tumor Diameter.

(B) and (C) Relative expression level of circPSD3 in the RT-qPCR cohort based on clinicopathological features, including lymph node metastasis (B), gender, age, multifocality, extrathyroidal extension (C). The experiments were repeated three times independently. Data are shown as the mean ± S.D. LN: lymph node.

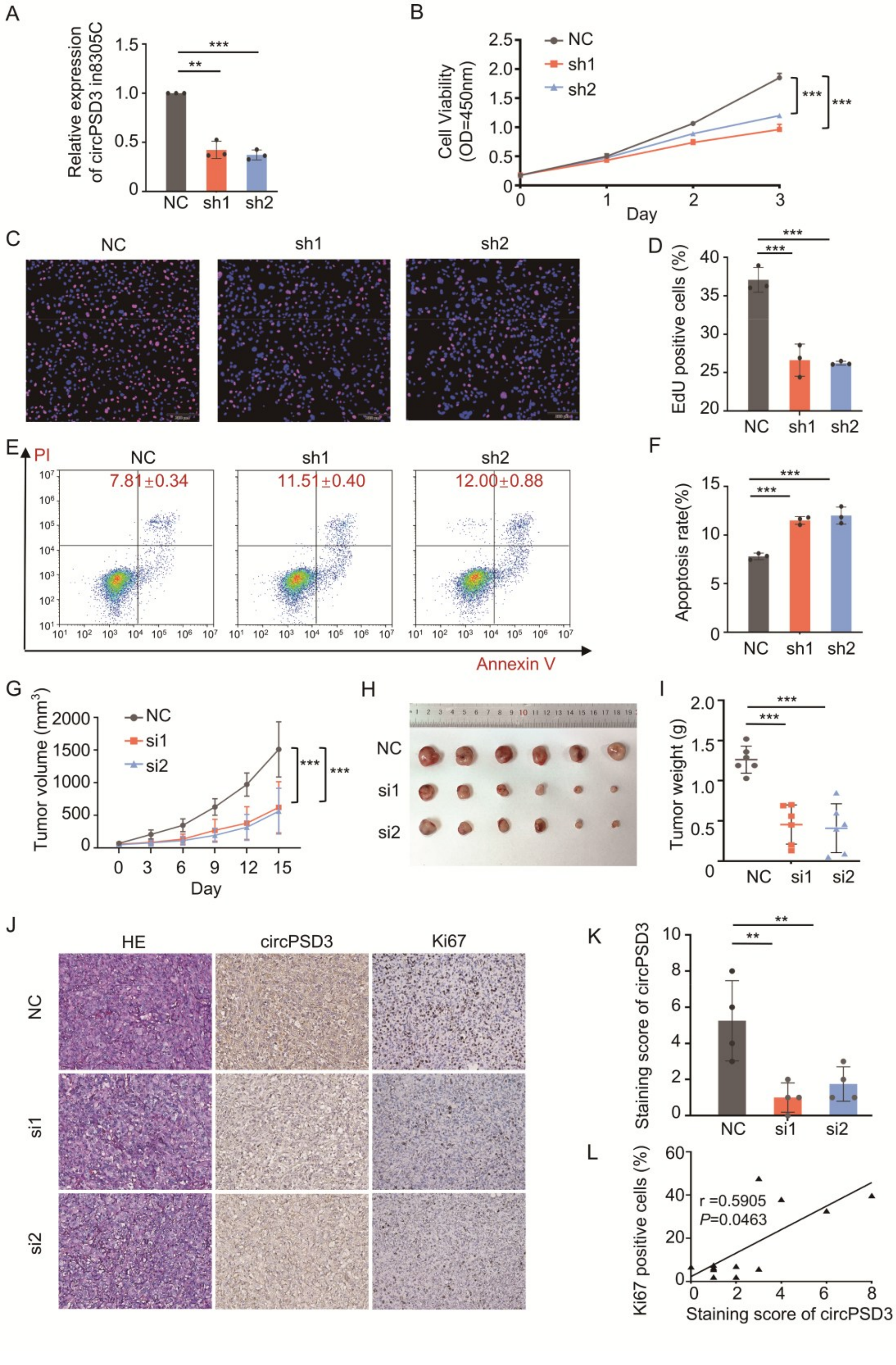

A
Relative expression of circPSD3 in8305C
NC
sh1
sh2
B
Cell Viability (OD=450nm)
Day
C
D
EdU positive cells (%)
E
PI
Annexin V
7.81±0.34
11.51±0.40
12.00±0.88
F
Apoptosis rate(%)
G
Tumor volume (mm3)
si1
si2
H
I
Tumor weight (g)
J
HE
circPSD3
Ki67
K
Staining score of circPSD3
L
Ki67 positive cells (%)
r =0.5905
P=0.0463
Staining score of circPSD3

Supplementary Figure 2: circPSD3 Enhances Cell Proliferation and Inhibits Apoptosis in Thyroid Carcinoma.

(A) The efficiency of circPSD3 knockdown in 8305C.

(B) Cell viability was detected by CCK-8 assay after the knockdown of circPSD3 in 8305C.

(C) Representative EdU fluorescence images demonstrated the inhibitory effect of circPSD3 on proliferation in 8305C.

(D) Statistical results of the EdU proliferation assay in 8305C.

(E) Apoptotic cells were analyzed by flow cytometry in 8305C.

(F) Statistical results of the apoptotic cell rate detected by flow cytometry in 8305C.

(G)-(I) Subcutaneous xenograft tumors after multiple intratumoral injections with siNC, sicircPSD3-1, and sicircPSD3-2. Tumor growth curves (G), gross appearance of tumors taken on the same scale (H), and tumor weight analysis (I) of 8305C.

(J) Representative images of 8305C xenograft tumors by HE, FISH, and Ki67 staining.

(K) The efficiency of circPSD3 knockdown in 8305C xenograft tumors by circPSD3 staining.

(L) Correlation between circPSD3 and Ki67 expression in 8305C xenograft tumors.

The experiments were repeated three times independently. Data are shown as the mean ± S.D. NC: negative control group. sh1: sh-circPSD3-1 group. sh2: sh-circPSD3-2 group.

*$P < 0.05$; **$P < 0.01$; ***$P < 0.001$.

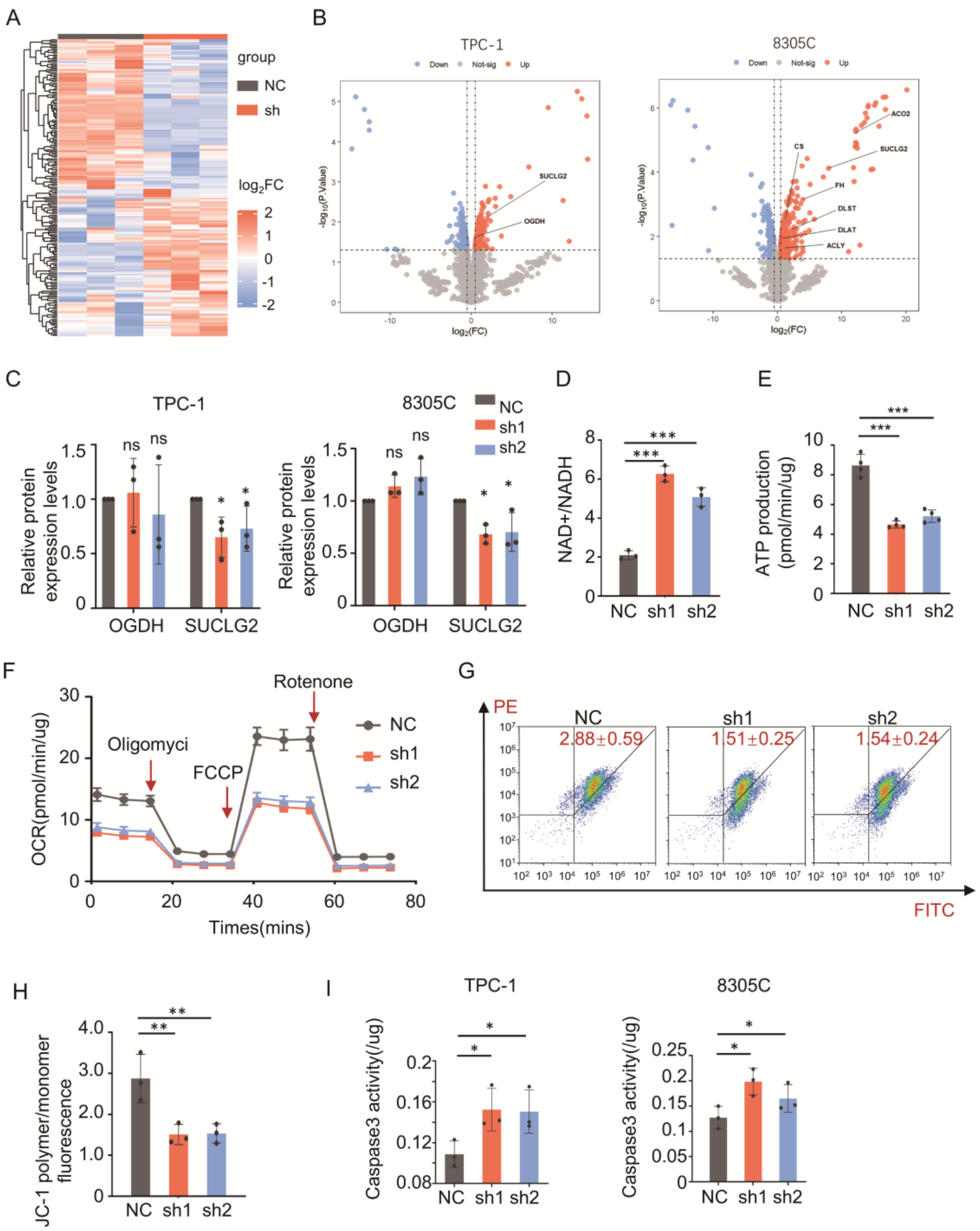

Supplementary Figure 3: circPSD3's Role in Regulating the TCA Cycle and Mitochondrial Function.

(A) Heatmap showing the differentially expressed protein profile between the sh-circPSD3 group and the NC group in 8305C.

(B) Volcano plot illustrates differentially expressed proteins between the two cell lines. Key enzymes of the TCA cycle that exhibit significant different expression level were highlighted.

(C) Statistical analysis of the grayscale intensity of protein stripes from Fig 3F, showing the expression levels of SUCLG2 was downregulated after circPSD3 knockdown while OGDH exhibited no significant difference.

(D) The NAD+/NADH ratio was elevated in the sh-circPSD3 group in 8305C.

(E) OCR was measured using a Seahorse XF analyzer in 8305C, indicating suppressed maximal

respiration and ATP production after circPSD3 knockdown.

(F) ATP production from OXPHOS was calculated based on OCR.

(G) MMP was measured by flow cytometry using JC-1. Red fluorescence (PE channel) represented polymers, and green fluorescence (FITC channel) represented monomers. Knockdown of circPSD3 led to a decrease in the polymer/monomer fluorescence intensity ratio, indicating mitochondrial depolarization.

(H) Statistical results of MMP. MMP is calculated as the JC-1 polymer/monomer fluorescence ratio.

(I) Caspase-3 was activated in the sh-circPSD3 group in 8305C.

The experiments were repeated three times independently. Data are shown as the mean ± S.D. MMP: mitochondrial membrane potential. OCR: oxygen consumption rate. NC: negative control group. sh1: sh-circPSD3-1 group. sh2: sh-circPSD3-2 group.

$*P < 0.05$; $**P < 0.01$; $***P < 0.001$.

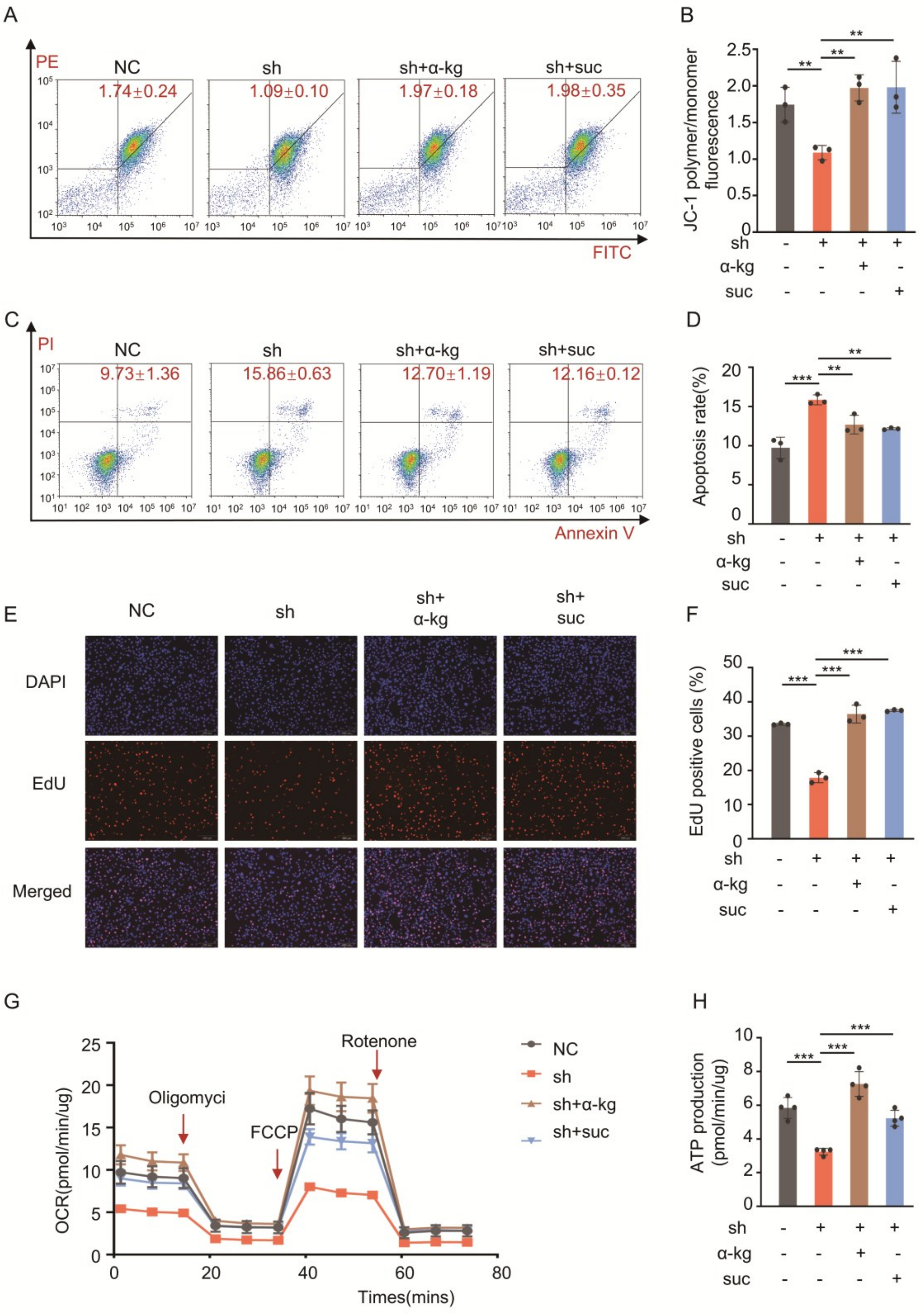
A
PE
NC
1.74±0.24
sh
1.09±0.10
sh+α-kg
1.97±0.18
sh+suc
1.98±0.35
FITC
B
JC-1 polymer/monomer fluorescence
sh
α-kg
suc
C
PI
NC
9.73±1.36
sh
15.86±0.63
sh+α-kg
12.70±1.19
sh+suc
12.16±0.12
Annexin V
D
Apoptosis rate(%)
E
NC
sh
sh+ α-kg
sh+ suc
DAPI
EdU
Merged
F
EdU positive cells (%)
G
OCR(pmol/min/ug)
Oligomyci
FCCP
Rotenone
Times(mins)
NC
sh
sh+α-kg
sh+suc
H
ATP production (pmol/min/ug)

Supplementary Figure 4: circPSD3 Promotes Cell Proliferation by Regulating the TCA Cycle in Thyroid Carcinoma.

(A) and (B) Flow cytometry and statistical results demonstrate the decreased MMP in the sh-circPSD3 group, rescued by the restoration of α-ketoglutarate and succinate in 8305C.

(C) and (D) Flow cytometry and statistical results show the increased apoptotic cell rate in the sh-circPSD3 group, rescued by the restoration of α-ketoglutarate and succinate in 8305C.

(E) and (F) Representative EdU fluorescence images and statistical results demonstrate the inhibitory effect on proliferation of circPSD3-knockdown, rescued by the restoration of α-ketoglutarate and succinate in 8305C.

(G) and (H) The suppressed OCR and decreased ATP production in the sh-circPSD3 group, rescued by the restoration of α-ketoglutarate and succinate in 8305C.

The experiments were repeated three times independently. Data are shown as the mean ± S.D. α-kg: α-ketoglutarate. suc: succinate. NC: negative control group. sh: sh-circPSD3 group.

*$P < 0.05$; **$P < 0.01$; ***$P < 0.001$.

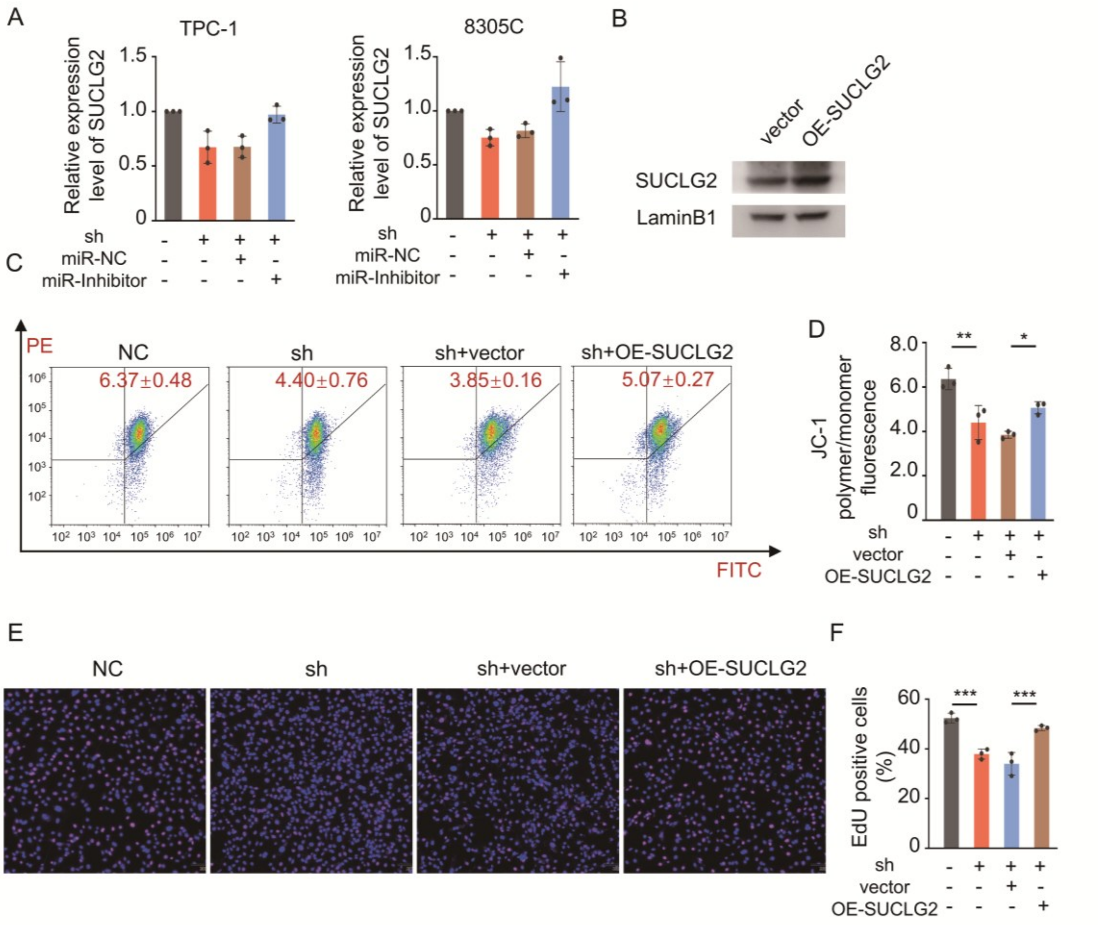

Supplementary Figure 5: circPSD3 Functions as a Sponge of miR-338-5p.

(A) Statistical analysis of the grayscale intensity of protein stripes from Fig 5I, showing the expression levels of SUCLG2 was rescued by miR-338-5p inhibitor after circPSD3 knockdown.

(B) Western blotting demonstrates the efficiency of overexpressing SUCLG2.

(C) and (D) Flow cytometry and statistical results demonstrate that overexpression of SUCLG2 rescues the decreased MMP caused by circPSD3 knockdown in 8305C.

(E) and (F) Representative EdU fluorescence images and statistical results demonstrate the inhibitory effect on proliferation of circPSD3-knockdown, rescued by SUCLG2 overexpression in 8305C.

The experiments were repeated three times independently. Data are shown as the mean ± S.D. NC: negative control group. sh: sh-circPSD3 group. *P < 0.05; **P < 0.01; ***P < 0.001.

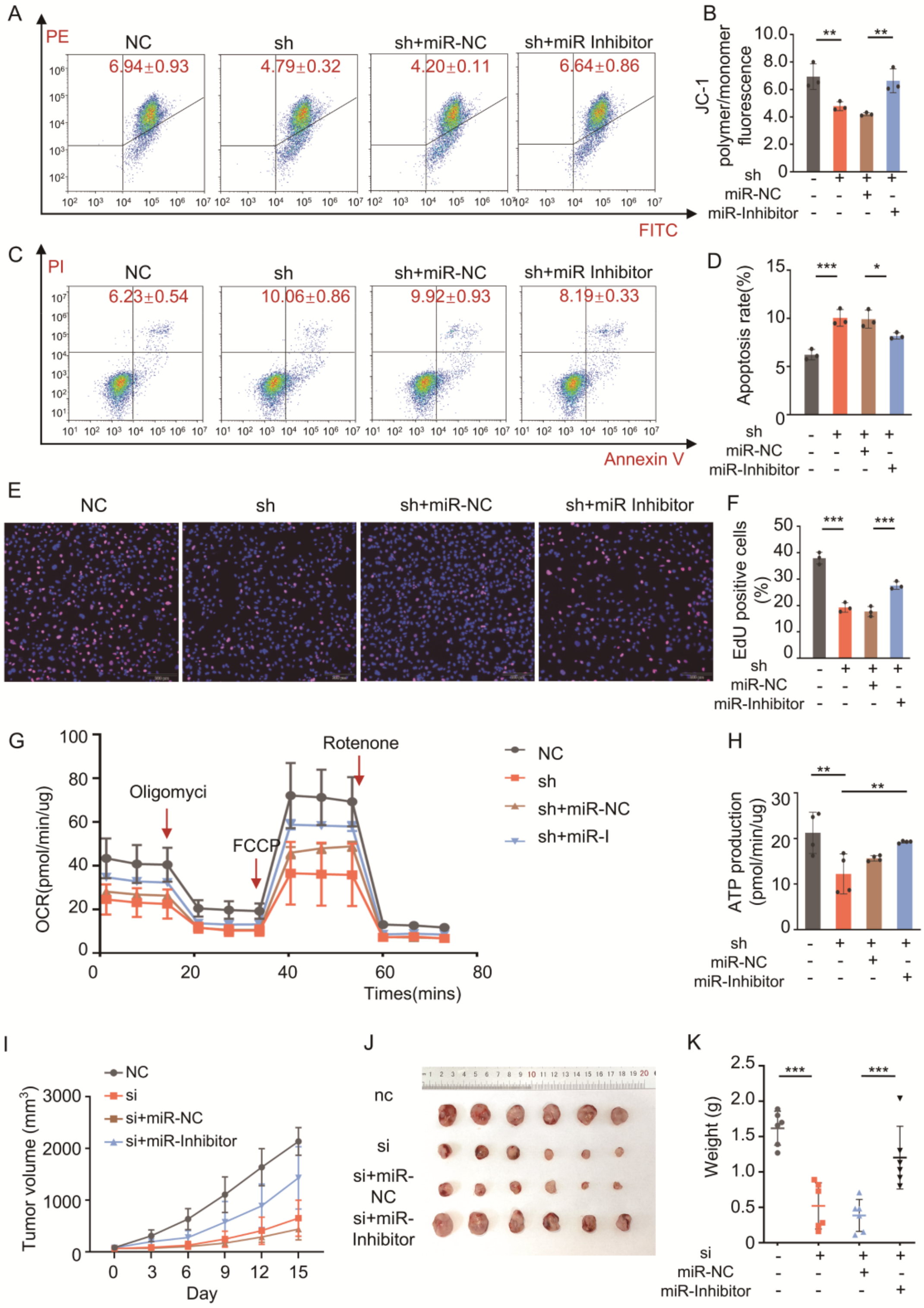
A
PE
NC
6.94±0.93
sh
4.79±0.32
sh+miR-NC
4.20±0.11
sh+miR Inhibitor
6.64±0.86
FITC
B
JC-1 polymer/monomer fluorescence
sh
miR-NC
miR-Inhibitor
C
PI
NC
6.23±0.54
sh
10.06±0.86
sh+miR-NC
9.92±0.93
sh+miR Inhibitor
8.19±0.33
Annexin V
D
Apoptosis rate(%)
E
NC
sh
sh+miR-NC
sh+miR Inhibitor
F
EdU positive cells (%)
G
OCR(pmol/min/ug)
Oligomyci
FCCP
Rotenone
Times(mins)
NC
sh
sh+miR-NC
sh+miR-I
H
ATP production (pmol/min/ug)
I
NC
si
si+miR-NC
si+miR-Inhibitor
Tumor volume (mm^3)
Day
J
nc
si
si+miR-NC
si+miR-Inhibitor
K
Weight (g)
si
miR-NC
miR-Inhibitor

Supplementary Figure 6: circPSD3 Regulates TCA Cycle and Promotes Proliferation by Sponging miR-338-5p

(A) and (B) Flow cytometry and statistical results reveal that the miR-338-5p inhibitor reverses the decreased MMP in the sh-circPSD3 group in 8305C.

(C) and (D) Flow cytometry and statistical results show that the miR-338-5p inhibitor reverses the increased apoptotic cell rate in the sh-circPSD3 group in 8305C.

(E) and (F) Representative EdU fluorescence images and statistical results reveal that the miR-338-5p inhibitor reverses the inhibitory effect on proliferation in the sh-circPSD3 group in 8305C.

(G) and (H) The suppressed mitochondrial respiration and ATP production in the sh-circPSD3 group, rescued by the miR-338-5p inhibitor in 8305C.

(I) Subcutaneous xenograft tumors after multiple intratumoral injections with siNC, si-circPSD3, si-circPSD3+NC-inhibitor, si-circPSD3+miR-338-5p inhibitor. Tumor growth curves (I), gross appearance of tumors taken on the same scale (J), and tumor weight analysis (K) of 8305C.

The experiments were repeated three times independently. Data are shown as the mean ± S.D. miR-NC: miRNA inhibitor Negative Control. miR-I: hsa-miR-338-5p inhibitor. NC: negative control group. sh: sh-circPSD3 group. *$P < 0.05$; **$P < 0.01$; ***$P < 0.001$.
